# Mortality Patterns and Site Heterogeneity of Severe Malaria in African Children

**DOI:** 10.1371/journal.pone.0058686

**Published:** 2013-03-07

**Authors:** Eric Kendjo, Tsiri Agbenyega, Kalifa Bojang, Charles R. J. C. Newton, Marielle Bouyou-Akotet, Florian Pedross, Maryvonne Kombila, Raimund Helbok, Peter Gottfried Kremsner

**Affiliations:** 1 Medical Research Unit, Albert Schweitzer Hospital, Lambaréné, Gabon; 2 Institute of Tropical Medicine, University of Tübingen, Tübingen, Germany; 3 Department of Parasitology, Mycology and Tropical Medicine, Faculty of Medicine, University of Health Sciences, Libreville, Gabon; 4 Université Pierre et Marie-Curie – CNR paludisme, Paris, France; 5 University of Science and Technology, School of Medical Science, Kumasi, Ghana; 6 Medical Research Council Laboratories, Banjul, The Gambia; 7 KEMRI Centre for Geographic Medicine Research (Coast), Kilifi, Kenya; 8 Institute of Child Health, University College London, London, United Kingdom; 9 Department of Medical Statistics, Informatics and Health Economics, Medical University Innsbruck, Innsbruck, Austria; 10 Clinical Department of Neurology, Innsbruck Medical University, Innsbruck, Austria; Universidade Federal do Acre (Federal University of Acre), Brazil

## Abstract

**Background:**

In this study we aimed to assess site heterogeneity of early, intermediate, and late mortality prediction in children with severe *Plasmodium falciparum* malaria in sub-Saharan Africa.

**Methods:**

Medical records of 26,036 children admitted with severe *Plasmodium falciparum* malaria in six hospital research centers between December 2000 to May 2005 were analyzed. Demographic, clinical and laboratory data of children who died within 24 hours (early), between 24 and 47 hours (intermediate) and thereafter (48 hours or later, late mortality) were compared between groups and survivors.

**Results:**

Overall mortality was 4·3% (N = 1,129). Median time to death varied across sites (*P*<0·001), ranging from 8h (3h–52h) in Lambaréné to 40h (10h–100h) in Kilifi. Fifty-eight percent of deaths occurred within 24 hours and intermediate and late mortality rate were 19% and 23%, respectively. Combining all sites, deep breathing, prostration and hypoglycemia were independent predictors for early, intermediate and late mortality (*P*<0·01). Site specific independent predictors for early death included prostration, coma and deep breathing at all sites (*P*<0·001). Site specific independent predictors for intermediate and late death largely varied between sites (*P*<0·001) and included between 1 and 7 different clinical and laboratory variables.

**Conclusion:**

Site heterogeneity for mortality prediction is evident in African children with severe malaria. Prediction for early mortality has the highest consistency between sites.

## Introduction

_ENREF_1Despite decreasing transmission rates, *Plasmodium falciparum* malaria remains an important health problem affecting approximately 200 million patients a year.[1] Mortality is especially high among young children in sub-Saharan Africa.[2]

Disease severity and morbidity strongly depend on patient-, disease-, and parasite-specific factors.[3], [4] Case fatality is associated with poor access to health services, delayed diagnosis, increased resistance of malaria parasites, co-infections, the use of ineffective drugs and delay_ENREF_9 in effective treatment.[5], [6] Mortality rate is highest in the first 24 hours of hospitalization, significantly decreases thereafter, and has been associated with pre-admission seizures, jaundice and deep breathing.[7], [8] Factors for intermediate (24–47h after admission) and late (>48h after admission) mortality have not been elucidated so far. Moreover, predictive factors for early, intermediate and late mortality may largely differ among sites.

In the present work we aim to explore site heterogeneity for mortality prediction in six sites in sub-Saharan Africa using multivariate models for early, intermediate and late mortality.

## Methods

### Study design

The Severe Malaria in African Children (SMAC) clinical research network is a cross-sectional observational database of patients hospitalized with malaria. The study used data from 26,296 consecutively hospitalized children, who presented with severe *P. falciparum* malaria to six hospital-based research units in sub-Saharan Africa between December 2000 and May 2005.

### Data collection

Data collection and quality control measures were processed at each site, and standard operating procedures were developed as previously described.[9], [10] A standardized case report form was used to collect data after consent was provided. Patients were followed throughout their hospitalization and, once the outcome was known, the completed form was submitted to the data entry team at each site. Data were double entered. The data collection system combined some data scrubbing tools to systematically examine data for flaws. An error file and queries were created for data outside predefined ranges. All discrepancies and queries were resolved manually by the clerks and data managers at each site; then the data from each site were to the data coordinator, who was responsible for maintaining the pooled database for the network and for ensuring its security. Informed consent was obtained from the accompanying parent or guardian. The demographic variables recorded were: age on admission, gender, site of the study and z-score. The weight-for-age (z-score) measurement that is a reflection of nutritional status was classified as follows: children with a Z score < -2 were classified as normal nourished, children with a Z score < -2 and > =  -3 as moderately malnourished, and children with a Z score < -3 as severely malnourished. The history of present illness variables were: convulsion and vomiting prior to admission. The laboratory variables were: hemoglobin, hematocrit, white blood cells (WBC), platelets, lactate and glucose. Clinical variables were: deep breathing,[11] irregular breathing, consciousness which was evaluated with the Blantyre Coma Score (BCS) evaluating motor function (0–2), verbal response (score 0– 2), and eye movements (score 0–1).[12] Prostration was defined by one of the four signs: not being able to breast-feed, to sit, to stand up, or to walk, depending on the age of the child.[13]

### Statistical analysis

We defined the time to death or discharge as the interval between the time of hospital presentation and the time of death or when the patient was discharged. We assigned all participants to one of four categories derived from time between admission and death: early (0–23 hours), intermediate (24–47 hours), late mortality (≥48 hours), and those discharged alive.

To investigate potential differences on admission in demographic, clinical and laboratory manifestations of severe falciparum malaria in different survival group, variables were summarized as frequencies, means (standard deviation), medians (interquartile range) and compared using chi-square test, Anova, or non-parametric testing (Kruskal-Wallis test) as appropriate. Bonferroni’s test was performed for multiple comparisons of time and site. The chi-square test for trend was used to identify the linear trend between early, middle and late mortality groups.

Mortality prediction: in the first step, we performed separate stepwise backward multivariable logistic regression analysis to assess the effect of predefined variables on early, intermediate and late mortality. Two-way interaction terms between variables were tested, but none were significant. We did not include variables with more than 5% missing data points and therefore had to exclude plasma lactate from all regression models. Due to the small proportion of subjects dying in Lambaréné, we combined data from both Gabonese sites. The effect estimates are the odds ratios with their 95% confidence interval for each of early, middle and late mortality compared with those discharged alive. In the second step site specific model were analyzed using the same variables and outcome measures. To validate our results, we have estimated the power of each logistic regression by running in G*POWER 3.1.5 package (Franz Faul, Universität Kiel, Germany), the post-hoc power calculation for beta-coefficents/odds ratios in logistic regression models for early, middle and late death by site with covariates. Statistical analysis was performed using Stata, version 12 (Stata Corp, College station, Tex). Information about a variable was “missing” if the case record did not mention the variable and was “unknown” if the case record mentioned that the variable was not tested for. Analyses resulting in values of *P*<0·05 were considered significant. All reported p-values are two-tailed.

## Results

Of the 26,296 children with severe malaria in six SMAC centers (Banjul, N  =  3,337; Blantyre, N  =  5,323; Kilifi, N  =  6,922; Kumasi, N  =  6,933; Lambaréné, N  =  1,794 and Libreville, N  =  1,727) 260 were excluded due to missing data, leaving 26,036 patients eligible for analysis. Demograhpics, clinical and admission laboratory findings were stratified by time to death and are illustrated in Table 1. There was not site specific difference for gender, however, as previously reported, age differed in general between sites (*P*<0.001, Kruskal Wallis rank test).

**Table 1:** Demographics, Clinical and Laboratory analyses.

	Time to death or discharged groups (hours)				
	0–23	24–47	≥48	Discharged Alive	Total
Number of patients	658	214	257	24,907	26,036
Demographic Characteristics					
Age (months)	29 (14–48)	25 (16, 43)	27 (15, 48)	25 (14, 46)	26 (14, 46)
Weight – for – Age Z-score	–2·2 (1·9)	–2·3 (1·8)	–3·2 (2·3)	–1·9 (1·7)	–1·9 (1·7)
Gender (Male)	334 (51)	129 (60)	127 (50)	13,424 (549)	14,014 (54)
Seizures prior to admission	330 (50)	118 (56)	76 (30)	7,797 (31)	8,321 (32)
Vomiting prior to admission	409 (63)	141 (67)	136 (53)	12,033 (48)	12,719 (49)
Examen Finding on Admission					
Deep breathing	327 (50)	93 (44)	72 (28)	2,327 (9)	2,819 (11)
Indrawing	254 (39)	70 (33)	80 (31)	2,655 (11)	3,059 (12)
Irregular Breathing	212 (33)	45 (21)	36 (14)	1,070 (4)	1,363 (5)
Prostration	619 (94)	200 (94)	218 (85)	14,833 (60)	15,870 (61)
Coma (Blantyre Coma Score ≤ 2)	334 (51)	86 (40)	60 (23)	1,955 (8)	2,435 (9)
Laboratory Tests on Admission					
Hyperparasitemia (parasitemia ≥250 000 p/µL)	149 (23)	57 (27)	38 (15)	4,715 (19)	4,959 (19)
Severe anemia (hematocrit<15% or haemoglobin<5 g/L)	232 (35)	55 (26)	69 (27)	5,023 (20)	5,379 (21)
Hypoglycemia (glucose<2·2 mmol/L)	141 (24)	42 (22)	45 (19)	876 (4)	1,104 (5)
Hyperlactatemia (lactate >5 mmol/L)*	379 (76)	95 (60)	61 (44)	4,961 (29)	5,496 (31)

Data are expressed as mean (SD), median (IQR) or number (%) unless otherwise indicated; Haemoglobin was not measured in Blantyre and lactate in Kilifi

Abbreviations: IQR, interquartile range; SD, standard deviation.

* missing values in 32%

### Time to death

**Figure 1:**
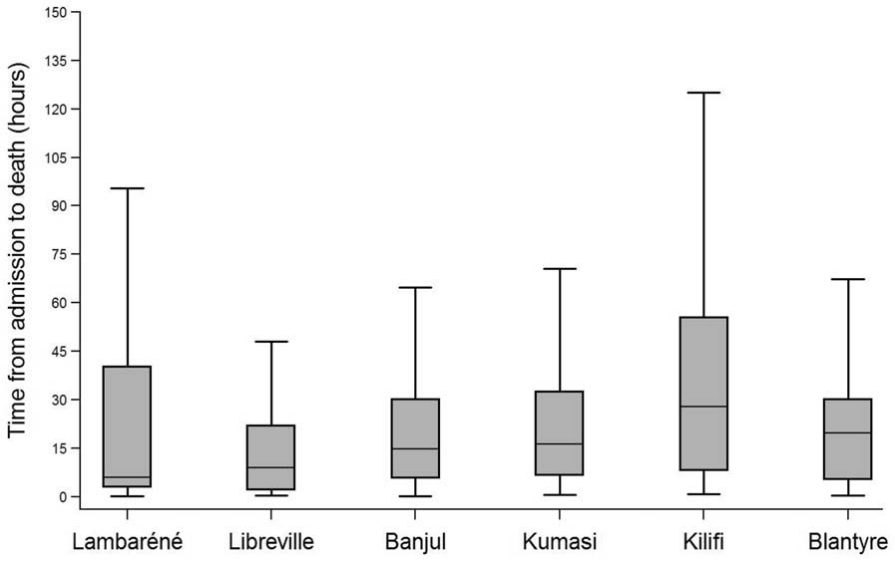
Boxplot analysis showing time to death by site representing median (line), interquartile range(box).

The overall median time to death was 19h (6–44h) and varied across sites, ranging from 8h (3–52h) in Lambaréné to 40h (10–104h) in Kilifi; (Libreville 10h (2–23h); Banjul 16h (6–32h); Kumasi 18h (7–36h); and Blantyre 20h (5–32h)). Time to death was significantly longer in Kilifi when compared to all other sites (P = 0.001, Bonferroni’s test). (Figure 1).

### Case fatality and site variability

**Figure 2:**
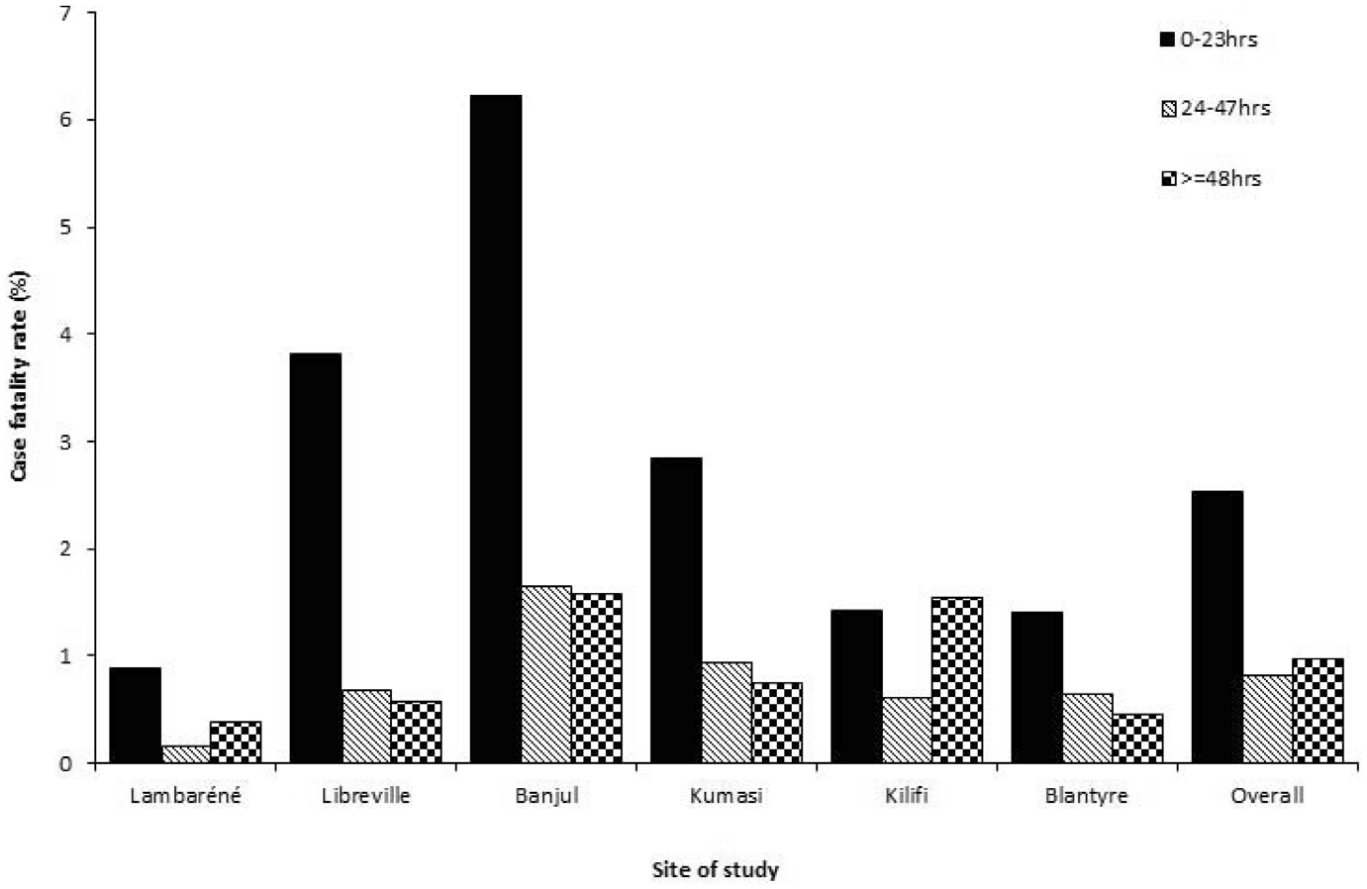
Bar graph showing the case fatality rate of severe falciparum malaria in 6 groups classified by time to death after admission. Case fatality rate was not similar in a time group and within a site (p<0.001). Case fatality rate significantly decreased with time in all sites except in Lambaréné and Kilifi.

Overall, mortality was 4·3% (N = 1,129/26,296); 58% (N = 658) of deaths occurred within 24h, 19% (N = 214) within 48h, and 23% (N = 257) after more than 47h (*P*<0·001). The early mortality was highest in Banjul (6·2%; *P*<0·001) (Figure 2).

Site specific mortality significantly decreased over time in all sites (*P*<0·001) except in Lambaréné and Kilifi (Figure 2).

### Demographic characteristics, clinical and laboratory features of severe malaria stratified by time to death

All clinical and laboratory test variables of children who survived differed from children who died early, between 24–47h and >48h after admission (*P*<0·001, Table 1). The proportion of admission variables including deep breathing, intercostal recessions, irregular breathing, coma, prostration, hypoglycemia, and hyperlactatemia significantly decreased in patients who died later (P<0·001), whereas the proportion of convulsions prior to admission, vomiting prior to admission, severe anemia, and hyperparasitemia did not show a specific trend with time to death.

Overall, z-score was associate to early, intermediate and late mortality (P<0·001). Site-specific analysis revealed a significant association between severe malnutrition and mortality over time in all site except in Lambaréné (P = 0·8) and Libreville (P = 0·09).

### Adjusted analyses for early, intermediate, and late mortality

#### All sites analysis

Results of a multivariate logistic regression analysis are shown in table 2. Variables used in the model were site, age, z-score, gender, convulsion prior to admission, vomiting prior to admission, deep breathing, intercostal recessions, irregular breathing, prostration, coma, hyperparasitemia, severe anemia and hypoglycemia. No interaction between variables was found. Combining all sites, deep breathing, prostration and hypoglycemia were independent predictors for early, intermediate and late mortality (*P*<0·01).

**Table 2:** Predictors for early, middle, late death in African children with severe falciparum malaria and stratified by country.

Times	Varibales	Banjul (N = 3,337)		Blantyre (N = 5,323)		Gabon (N = 3,521)		Killifi (N = 6,922)		Kumasi (N = 6,933)		Overall (N = 26,036)	
		OR(95% CI)	p	OR(95% CI)	p	OR(95% CI)	p	OR(95% CI)	p	OR(95% CI)	p	OR(95% CI)	p
case fatality		316 (9.4)		135 (2.5)		114 (3.2)		249 (3.6)		315 (4.5)		1,129 (4.3)	
Early *vs* Alive													
Case fatality		208/3,229 (6.4)		75/5,263 (1.4)		82/3,489 (2.3)		97/6,770 (1.4)		196/6,814 (2.9)		658/25,565 (2.6)	
	Gender (male)	0·6 (0·4–0·9)	0·02	–	–	–	–	–	–	–	–	–	–
	Age < 5 years	–	–	–	–	–	–	–	–	0·6 (0·4–0·8)	0·006	–	–
	Moderately malnourished	–	–	–	–	–	–	–	–	1·5 (1·0–2·2)	0·08	1·2 (0·9–1·5)	0·3
	Severely malnourished	–	–	–	–	–	–	–	–	1·8 (1·2–2·7)	0·005	1·4(1·1–1·8)	<0·01
	Convulsions prior to admission	–	–	–	–	–	–	0·4 (0·2–0·8)	0·004	0·7 (0·5–1·0)	0·03	–	–
	Intercostal recessions	–	–	–	–	–	–	1·9 (1·1–3·1)	0·01	–	–	–	–
	Irregular breathing	2·8 (1·7–4·6)	<0·001	–	–	3·1 (1·6–6·2)	0·001	3·1 (1·6–6·0)	0·0006	2·5 (1·7–3·8)	<0·001	2·4(1·9–3·0)	<0·001
	Vomiting prior to admission	–	–	1·8 (1·1–3·2)	0·03	–	–	–	–	–	–	–	–
	Deep breathing	2·0 (1·2–3·4)	0·007	3·5 (1·9–6·3)	<0·001	4·3 (2·3–8·1)	<0·001	4·1 (2·4–7·1)	<0·001	3·8 (2·6–5·4)	<0·001	3·3(2·6–4·1)	<0·001
	Prostration	4·6 (2·8–7·5)	<0·001	5·7 (2·4–13·5)	0·0001	11·6 (4·0–33·8)	<0·001	5·2 (2·3–11·7)	0·0001	2·3 (1·4–3·9)	0·0009	4·6(3·4–6·2)	<0·001
	Coma	2·3 (1·5–3·6)	0·0003	5·9 (3·2–10·6)	<0·001	2·9 (1·5–5·3)	0·0008	4·1 (2·3–7·3)	<0·001	4·6 (3·1–6·9)	<0·001	3·9(3·1–4·9)	<0·001
	Severe anemia			2·6 (1·5–4·7)	0·001	–	–	–	–	–	–	–	–
	Hypoglycemia	–	–	3·0 (1·4–6·4)	0·004	3·2 (1·6–6·5)	0·0008	3·0 (1·7–5·3)	0·0002	4·0 (2·6–6·2)	<0·001	3·3(2·6–4·3)	<0·001
	Hyperparasitemia	3·6 (1·8–7·5)	0·0004	–	–	–	–	–	–	–	–	–	–
Middle *vs* Alive													
Case fatality		57/3,078(1.9))		35/5,223(0.7)		16/3,423(0.5)		43/6,716(0.6)		63/6,681(0.9)		214/25,121(0.9)	
	Moderately malnourished	–	–	–	–	–	–	–	–	–	–	1·0(0·6–1·5)	0·8
	Severely malnourished	–	–	–	–	–	–	–	–	–	–	1·5(1·0–2·1)	<0·05
	Convulsions prior to admission	5·3 (2·0–14·1)	0·0008	2·6 (1·2–5·6)	0·02	–	–	–	–	–	–	–	–
	Vomiting prior to admission	–	–	3·2 (1·5–7·1)	0·004	–	–	2·2 (1·1–4·3)	0·02	–	–	2·0(1·4–2·8)	<0·001
	Intercostal recessions	–	–	6·9 (3·0–16·2)	<0·001	–	–	–	–	–	–	–	–
	Irregular breathing	–	–	–	–	–	–	3·5 (1·3–9·3)	0·01	2·6 (1·3–5·2)	0·007	–	–
	Deep breathing	5·4 (2·5–12·0)	<0·001	–	–	–	–	3·1 (1·4–6·5)	0·004	3·9 (2·1–7·1)	<0·001	3·6(2·6–5·1)	<0·001
	Prostration	–	–	4·4 (1·6–11·6)	0·003	10·1 (2·2–46·1)	0·003	4·5 (1·9–10·3)	0·0004	–	–	2·8(1·8–4·3)	<0·001
	Coma	–	–	–	–	–	–	–	–	3·7 (2·0–6·7)	<0·001	2·9(2·0–4·1)	<0·001
	Hypoglycemia	4·4 (1·8–11·0)	0·002	3·6 (1·3–9·9)	0·01	–	–	4·6 (2·1–9·9)	0·0001	2·8 (1·3–5·9)	0·007	3·5(2·3–5·2)	<0·001
Late *vs* Alive													
Case fatality		51/3,072 (1.7)		25/5,213(0.5)		16/3,423(0.5)		109/6,782(1.6)		56/6,674(0.8)		257/25,164(1.0)	
	Age < 5 years	–	–	–	–	–	–	–	–	0·4 (0·2–0·7)	0·002	–	–
	Moderately malnourished	1·0 (0·4–2·8)	0·9	–	–	–	–	3·5 (1·7–7·3)	0·0007	1·7 (0·8–3·5)	0·1	1·7(1·1–2·5)	<0·01
	Severely malnourished	3·0 (1·4–6·4)	0·005	–	–	–	–	7·5 (3·9–14·3)	<0·001	1·7 (0·8–3·4)	0·1	3·2(2·4–4·5)	<0·001
	Convulsions prior to admission	–	–	–	–	–	–	–	–	0·4 (0·2–0·9)	0·02	–	–
	Intercostal recessions	–	–	–	–	–	–	2·0 (1·2–3·1)	0·004	3·0 (1·6–5·7)	0·0008	1·7(1·2–2·5)	<0·01
	Irregular breathing	–	–	–	–	–	–	3·2 (1·5–6·7)	0·002	2·9 (1·3–6·5)	0·009	–	–
	Deep breathing	–	–	–	–	–	–	–	–	–	–	1·7(1·2–2·5)	<0·01
	Prostration	3·1 (1·5–6·7)	0·003	5·0 (1·9–13·1)	0·001	–	-	2·1 (1·3–3·2)	0·001	2·3 (1·2–4·5)	0·01	2·3(1·7–3·2)	<0·001
	Coma	–	–	–	–	10·7 (3·9–29·0)	<0·001	–	–	–	–	1·6(1·1–2·4)	<0·05
	Hypoglycemia	–	–	6·9 (2·2–21·3)	0·0008	–	–	6·2 (3·7–10·2)	<0·001	2·6 (1·1–6·2)	0·03	3·5(2·4–5·2)	<0·001
	Hyperparasitemia	–	–	–	–	–	–	0·4 (0·2–0·9)	0·01	–	–	–	–

Abbreviations: CI = confidence interval; OR = odds ratio; P = P-value.

#### Site by site analysis

Deep breathing, prostration and coma were associated with early hospital death in all sites (*P*<0·001) *(* *Table 2*). Site specific independent predictors for intermediate and late death largely varied between sites and included between 1 and 7 different clinical and laboratory variables *(* *Table 2* *)*.

## Discussion

In this study we show that prediction of mortality in African children with severe malaria differs between sites. The most consistent predictive model included 3 admission variables, namely deep breathing, prostration and coma for death within 24 hours. Predictive models for intermediate and late mortality were highly variable between sites.

Mortality was highest in the first 24 hours after admission, which is well described in *Plasmodium falciparum* malaria.[7], [14] Clinical signs and symptoms of patients who die early usually comprise abnormalities in the respiratory and central nervous system.[15] A combination of deep breathing, irregular breathing and prostration or coma should prompt the clinician to supply immediate and appropriate care to the patient,[13] as delay in effective treatment is associated with poor prognosis.[5], [6], [16], [17], [18] Interestingly, we found that clinical and laboratory findings predicting intermediate and late mortality were less prevalent on admission. Therefore daily reevaluation seems necessary to identify children at risk for pending deterioration and fatal course.[13]

Median time to death significantly differed from site to site: Site specific uncontrollable factors contributing to this finding may be the transmission intensities and seasonality at each site.[19] Other pre-hospital factors including educational status and access to appropriate clinics may also play an important role but are potentially changeable.

Multivariate analysis predicting early, intermediate and late mortality at all sites were prostration, deep breathing and hypoglycaemia. This finding underlines the importance of neurologic manifestations of *P. falciparum* related mortality. Cerebral malaria is still associated with a high mortality even when appropriate therapy and optimal care are provided.[20] Impaired consciousness, prostration and seizures often precipitate coma and may lead to metabolic acidosis commonly presenting with respiratory distress.[15] Hypoglycemia is a main complication in children with severe malaria and is associated with poor outcome.[21]

Lactate concentrations is a known predictor for poor outcome in *Plasmodium falciparum* malaria.[22] Increased lactate production is commonly associated with metabolic acidosis and hyperventilation in children.[23] Unfortunately, we could not further elaborate on this prognostic marker due to the high number of missing plasma lactate levels in our study population.

Malnutrition is also a risk factor for death in hospitalized African children and is considered to be the underlying cause of more than 50% of all childhood deaths in the world.[24] It is associated with malaria case fatality rate, time to death or discharged in Banjul and Kilifi like observed in other malaria endemic areas.[25] Malnutrition and malaria share certain consequences, including cognitive impairment and decreased school performance; whether and how malnutrition influences malaria morbidity remains unknown.[26], [27]

We observed a large variability of predictors for intermediate and late mortality between sites. It has been reported, that various manifestations of severe malaria have different predictablity for mortality at different sites.[10] The patterns of severe disease are different between sites reflecting disparate patient and parasite populations as well as different admission criteria and health care systems but also variations in transmission and seasonality.[28] Another explanation may be the frequency of cerebral malaria varying according to malaria endemicity and transmission.[28] In The Gambia, with a highly seasonal malaria transmission, cerebral malaria was more prevalent than severe anemia.[21] This depends on the causes of the illness, resources available, cost and availability of drugs. In many parts of Africa, children are often brought to hospital after several hours or even days of prolonged or recurrent convulsions.[12]

The limitations of this study have been previously well described [12] _ENREF_19_ENREF_19_ENREF_19_ENREF_19 and include the _ENREF_17_ENREF_17_ENREF_17_ENREF_11_ENREF_11_ENREF_11lack of information about exact time of disease onset, about the distance from home to health centers, description of site specific medical care for the patients and the large number of missing plasma lactate levels. Including these variables could have influenced the overall analysis and in specific the number of parameters found to be associated to early, intermediate and late mortality at each site. Furthermore, subanalysis for middle mortality in Gabon, Blantyre and Kilifi and for late mortality in Gabon and Blantyre suffers of low statistical power due to the low mortality rate at these sites. Therefore, the number of variables being associated in these models should be interpreted with caution.

In conclusion, different features of severe malaria were strongly associated with time to death in the largest multicentre clinical trial in Africa. Patients with severe malaria who died earlier have more frequently the life threatening symptoms of severe malaria than those who died later. However, important site differences exist with regards to clinical and biological manifestations of malaria in spite of the quality control measures installed and standard operating procedures developed at each site. These findings reinforce the continued attention that needs to be devoted to the early identification of patients at risk for severe malaria, the application of timely and effective treatment strategies and reevaluation of clinical symptoms and signs during hospitalization.
